# NOD2 and Toll-Like Receptors Are Nonredundant Recognition Systems of *Mycobacterium tuberculosis*

**DOI:** 10.1371/journal.ppat.0010034

**Published:** 2005-11-25

**Authors:** Gerben Ferwerda, Stephen E Girardin, Bart-Jan Kullberg, Lionel Le Bourhis, Dirk J. de Jong, Dennis M. L Langenberg, Reinout van Crevel, Gosse J Adema, Tom H. M Ottenhoff, Jos W. M. Van der Meer, Mihai G Netea

**Affiliations:** 1 Department of Internal Medicine, Radboud University Medical Center, Nijmegen, The Netherlands; 2 Nijmegen University Center for Infectious Diseases, Nijmegen, The Netherlands; 3 Unité de Pathogénie Microbienne Moléculaire, INSERM U389, Institut Pasteur, Paris Cedex, France; 4 Department of Gastroenterology, Radboud University Medical Center, Nijmegen, The Netherlands; 5 Department of Immunohematology and Blood Transfusion, Leiden University Medical Center, Leiden, The Netherlands; 6 Department of Tumor Immunology, Radboud University Medical Center, Nijmegen, The Netherlands; UCLA Research and Education Institute, United States of America

## Abstract

Infection with *Mycobacterium tuberculosis* is one of the leading causes of death worldwide. Recognition of *M. tuberculosis* by pattern recognition receptors is crucial for activation of both innate and adaptive immune responses. In the present study, we demonstrate that nucleotide-binding oligomerization domain 2 (NOD2) and Toll-like receptors (TLRs) are two nonredundant recognition mechanisms of *M. tuberculosis*. CHO cell lines transfected with human TLR2 or TLR4 were responsive to *M. tuberculosis*. TLR2 knock-out mice displayed more than 50% defective cytokine production after stimulation with mycobacteria, whereas TLR4-defective mice also released 30% less cytokines compared to controls. Similarly, HEK293T cells transfected with NOD2 responded to stimulation with *M. tuberculosis*. The important role of NOD2 for the recognition of *M. tuberculosis* was demonstrated in mononuclear cells of individuals homozygous for the *3020insC NOD2* mutation, who showed an 80% defective cytokine response after stimulation with *M. tuberculosis*. Finally, the mycobacterial TLR2 ligand 19-kDa lipoprotein and the NOD2 ligand muramyl dipeptide synergized for the induction of cytokines, and this synergism was lost in cells defective in either TLR2 or NOD2. Together, these results demonstrate that NOD2 and TLR pathways are nonredundant recognition mechanisms of *M. tuberculosis* that synergize for the induction of proinflammatory cytokines.

## Synopsis

Tuberculosis is one of the most prevalent infections worldwide, with 2 billion people believed to be infected, and 2 million deaths each year. In addition to representing a major health care problem in developing countries, concern is also growing about the increased incidence of tuberculosis in developed countries, especially in immunocompromised patients such as those with AIDS, transplantation, and immunosuppressive therapy. The present study describes the pathways that enable leukocytes to recognize *M. tuberculosis,* and demonstrates for the first time that NOD2, member of a new class of intracellular receptors, is an independent recognition mechanism for mycobacteria. NOD2 acts together with the earlier-described Toll-like receptors for the activation of host defenses during the encounter of leukocytes with *M. tuberculosis*. Understanding the mechanisms through which the cells of the immune system recognize *M. tuberculosis* can be an important step in designing new therapeutic approaches, as well as improving the limited success of current vaccination strategies.

## Introduction

Worldwide, 2 billion people are currently believed to be infected with *Mycobacterium tuberculosis,* with an estimated death toll of 2 million patients each year [1]. *M. tuberculosis* is an intracellular pathogen capable of infecting and surviving within the host's mononuclear cells (MNCs), and a coordinated response of the innate and adaptive immune systems is required for an effective host defense. This involves sequestration of the microorganism in macrophages within organized granulomas, and elimination of the pathogen through a combination of killing mechanisms and apoptosis of host macrophages [2]. These responses are coordinated by T helper 1-type proinflammatory cytokines, which are synthesized by phagocytes upon recognition of pathogen-associated molecular patterns of mycobacteria by pattern recognition receptors (PRRs).

Toll-like receptors (TLRs) are believed to be an important pattern recognition system of *M. tuberculosis*. A soluble, heat-stable mycobacterial fraction was initially reported to signal through TLR2, whereas heat-labile components associated with the cell wall were reported to signal through TLR4 [3]. Later, several components of mycobacteria were identified as being responsible for TLR2-dependent activation: the 19-kDa lipoprotein [4], lipomannan [5], phosphatidyl-myo-inositol mannoside [6], but not mannosyl-caped lipoarabinomannan from virulent *M. tuberculosis,* which mainly has anti-inflammatory effects through its interaction with the mannose receptor and dendritic cell-specific intercellular adhesion molecule 3-grabbing nonintegrin, also termed DC-SIGN [7,8]. Despite an abundance of in vitro data regarding the recognition of mycobacterial structures by TLR2 and TLR4, knock-out mice deficient for these receptors display remarkably little enhanced susceptibility to infection with *M. tuberculosis*. *TLR4* ^−/−^ mice showed variable responses to the challenge with *M. tuberculosis,* with either normal resistance to infection [9,10] or chronic pneumonia and increased mortality [11,12]. In one study, *TLR2* ^−/−^ mice had a decreased clearance of the bacteria and developed chronic pneumonia when infected with a low dose of microorganisms [13], whereas in other studies only minor effects have been found [9,14]. The role of TLRs in the recognition of mycobacteria was further highlighted in mice deficient for MyD88 (an adaptor molecule shared by almost all the receptors of the TLR family), which are highly susceptible to *M. tuberculosis* infection [15].

*Nucleotide-binding oligomerization domain 2 (NOD2),* initially described as a susceptibility gene for Crohn's disease [16,17], is an intracellular protein containing leucine-rich repeats (LRRs) similar to those found in TLRs. NOD2 is the PRR responsible for recognition of bacterial peptidoglycans from both gram-positive and gram-negative bacteria, through its interaction with muramyl dipeptide (MDP) [18]. In contrast, NOD1 recognizes peptidoglycans of gram-negative bacteria only [19]. The intracellular localization of both NOD2 and *M. tuberculosis,* the cell wall of which contains peptidoglycans, makes NOD2 a highly suitable candidate for the recognition of mycobacteria. Our results support the hypothesis that TLRs and NOD2 represent two nonredundant recognition systems of *M. tuberculosis,* and that an efficient activation of innate immunity requires both classes of receptors.

## Results

### The Role of TLR2 and TLR4 in the Recognition of *M. tuberculosis*

**Figure 1:**
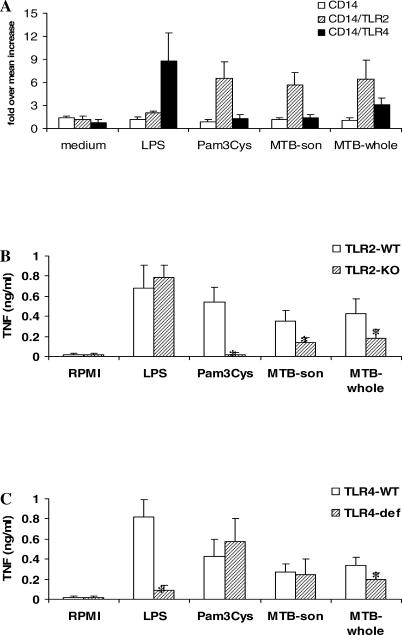
TLR2 and TLR4 Are Partially Responsible for Induction of Cytokine Production by *M. tuberculosis* (A) CHO cells cotransfected with CD14 and TLR2 (CD14/TLR2, hatched bars) induced potent expression of CD25 on the cell membrane as measured by FACS analysis, after stimulation with both a sonicated *M. tuberculosis* (MTB-son, 1 μg/ml) or whole mycobacteria (MTB-whole, 1 × 10^7^/ml). In contrast, cells transfected with CD14 and TLR4 (CD14/TLR4, black bars) were only moderately activated by whole *M. tuberculosis,* but not by the sonicated material. LPS (1 μg/ml) and Pam3Cys (5 μg/ml) served as control stimuli for TLR4 and TLR2, respectively. As controls, cells transfected only with CD14 were used (white bars). (B and C) Stimulation of TNF production by *M. tuberculosis* in peritoneal macrophages of mice deficient for TLR2 (TLR2-KO) (B) or TLR4 (TLR4-def) (C). Groups of five mice were stimulated with the indicated reagents, and the experiment was repeated twice. Medium-stimulated cells resulted in cytokine concentrations below detection limit. Data are presented as mean ± SD (*n* = 5; *p < 0.05).

TLR2 and TLR4 have been suggested to recognize bacterial structures of *M. tuberculosis* [20]. Indeed, a sonicate of *M. tuberculosis* strongly activated a Chinese hamster ovary fibroblast (CHO) cell line cotransfected with human TLR2 and CD14, whereas cells transfected with CD14 alone or a combination of CD14 and TLR4 displayed no signaling upon activation with the sonicated mycobacterial preparation (Figure 1A). However, when cells were stimulated with a preparation of whole mycobacteria, both TLR2 and TLR4-transfected cells were activated, although TLR2 activation was stronger (Figure 1A).

In line with these data, macrophages isolated from *TLR2* ^−/−^ mice displayed a 50%–75% reduction in tumor necrosis factor (TNF) production after stimulation with both *M. tuberculosis* preparations (Figure 1B), whereas TLR4-deficient macrophages showed a 30%–40% reduction of TNF release only when stimulated with the whole mycobacteria (Figure 1C). To confirm the role of TLR4 in the stimulation of cytokines by *M. tuberculosis,* we stimulated human MNCs with the whole mycobacterial preparation in the absence or presence of 10 μg/ml of a blocking anti-TLR4 antibody. TLR4 blockade completely inhibited lipopolysaccharide (LPS)-induced TNF secretion, and reduced *M. tuberculosis*-induced TNF secretion from 0.9 ± 0.2 to 0.5 ± 0.2 ng/ml (*p* < 0.05). These data confirm the role played by TLR2 and TLR4 in the recognition of *M. tuberculosis;* however, the significant remaining production of cytokines induced by *M. tuberculosis* in TLR2^−/−^ or TLR4-defective mice points to the presence of additional signaling pathway(s) for cytokine induction.

### The Role of Intracellular Recognition Systems in the Recognition of *M. tuberculosis*

**Figure 2:**
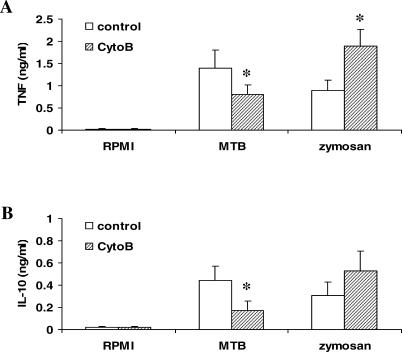
Blockade of Internalization of *M. tuberculosis* Impairs Recognition and Cytokine Production Blockade of *M. tuberculosis* internalization by cytochalasin B (20 μg/ml) impairs TNF (A) and IL-10 (B) stimulation in human MNCs stimulated with sonicated *M. tuberculosis* (1 × 10^6^ microorganisms/ml), but not zymosan (1 μg/ml). Data are presented as mean ± SD (*n* = 5; *p < 0.05).

The role of internalization in cytokine induction by *M. tuberculosis* was assessed by blocking it with cytochalasin B, an inhibitor of actin polymerization. Blocking internalization of *M. tuberculosis* partially inhibited *M. tuberculosis-*induced cytokine release in freshly isolated human MNCs (Figure 2). In contrast, cytochalasin B increased the zymosan-induced cytokine production in these MNCs, which likely results from prolonged stimulation of receptors at the cell surface by zymosan. The difference in the effect of cytochalasin B on *M. tuberculosis-* or zymosan-induced cytokines strongly suggests that internalization of *M. tuberculosis* is important for the induction of cytokine production through intracellular receptors.

### The Role of NOD1 and NOD2 in the Recognition of *M. tuberculosis*

**Figure 3:**
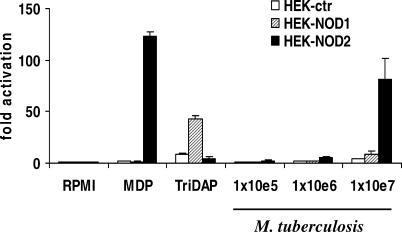
Stimulation of HEK Cells Transfected with NOD1 or NOD2 HEKs transfected with *NOD2* were strongly activated by whole *M. tuberculosis,* in contrast to cells transfected with *NOD1,* which were only weakly activated by *M. tuberculosis*. MDP (100 nM) and TriDAP (100 nM) served as control stimuli for NOD2 and NOD1, respectively. Data are presented as fold increase over unstimulated cells (mean ± SD).

Next, human embryonic kidney 293T cells (HEK293Ts) were transfected with either NOD1 or NOD2 expression vectors, and the ability of *M. tuberculosis* sonicates to activate these pathogen recognition receptors was followed by monitoring the level of NOD-dependent activation of a nuclear factor κB (NF-κB)-driven luciferease reporter gene. Strikingly, *M. tuberculosis* sonicates efficiently stimulated *NOD2,* while *NOD1*-transfected cells responded only modestly to the bacteria (Figure 3).

**Figure 4:**
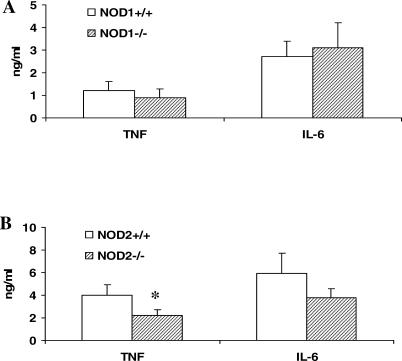
Stimulation of Cells from Mice Deficient in NOD1 or NOD2 Stimulation of cytokine production by sonicated *M. tuberculosis* in peritoneal macrophages of mice deficient for either NOD1 (A) or NOD2 (B). Groups of four mice were stimulated; medium-stimulated cells resulted in cytokine concentrations below detection limit. Data are presented as mean ± SD (*p < 0.05).

To investigate further the role of NOD1 and NOD2 in the recognition of *M. tuberculosis,* we stimulated peritoneal macrophages from *NOD1* ^−/−^ or *NOD2* ^−/−^ mice. We observed that peritoneal macrophages from *NOD2* ^−/−^ mice produced significantly less TNF than did control cells, supporting a role of NOD2 in the recognition of *M. tuberculosis* (Figure 4). In contrast, macrophages from *NOD1* ^−/−^ mice responded normally to *M. tuberculosis* (Figure 4), although as a control, their response to the NOD1 ligand FK156 was abrogated (unpublished data). Together, these results strongly support the notion that NOD2 represents an intracellular recognition system for *M. tuberculosis*.

### Stimulation of Cells from Patients with Defective NOD2 Recognition

**Figure 5:**
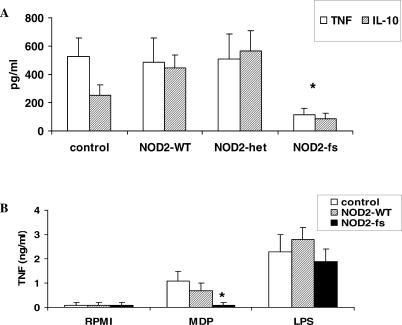
Human NOD2 Is a Receptor for *M. tuberculosis* (A) MNCs isolated from four patients with Crohn's disease homozygous for the *3020insC NOD2* mutation (NOD2fs), five patients heterozygous for *NOD2* mutations (NOD2het), five patients with the wild-type *NOD2* allele (NOD2WT), and five healthy volunteers with wild-type *NOD2* (control) were stimulated with 10 μg/ml sonicated *M. tuberculosis.* TNF (white bars) and IL-10 (black bars) were measured after 24 h of stimulation at 37 °C by specific RIA and ELISA, respectively. (B) MNCs isolated from four patients with Crohn's disease homozygous for the *3020insC NOD2* mutation (NOD2fs, black bars), five patients with the wild-type *NOD2* allele (NOD2WT, gray bars), and five healthy volunteers with wild-type *NOD2* (control), were stimulated with 1 μg/ml MDP or 10 ng/ml LPS*.* TNF were measured after 24 h of stimulation at 37 °C by specific RIA. Medium-stimulated cells resulted in cytokine concentrations below detection limit. Data are presented as mean ± SD (*p < 0.05).

In line with the hypothesis that NOD2 is involved in the recognition of mycobacteria, MNCs isolated from patients homozygous for the *3020insC* mutation synthesized 65%–80% less cytokines after stimulation with *M. tuberculosis* than did Crohn's disease patients heterozygous for the mutation or patients and volunteers homozygous for the wild-type variant (Figure 5A). This demonstrates that NOD2 is critical for the recognition of *M. tuberculosis* cell wall. In control experiments, cells from patients homozygous for the *3020insC* mutation were defective in their response to MDP, but not LPS (Figure 5B), as previously described [21].

### TLR2 and NOD2 Synergize for the Induction of Cytokine Release

**Figure 6:**
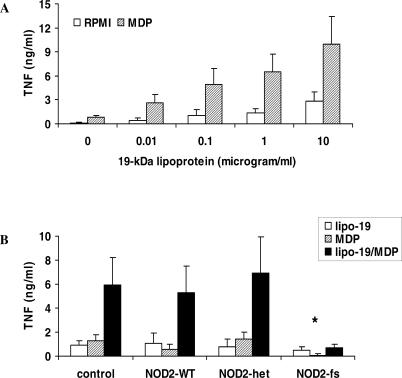
NOD2 and TLR2 Have Synergistic Effects on Cytokine Production (A) MNCs isolated from five healthy volunteers with wild-type *NOD2* were costimulated with 1 μg/ml MDP and increasing concentrations of 19-kDa lipoprotein (indicated on the x-axis). TNF was measured after 24 h of stimulation at 37 °C by specific RIA. (B) The synergistic effects observed in the five control volunteers, as well as in five Crohn's disease patients heterozygous for *NOD2* mutations (NOD2-het), and in five patients with the wild-type *NOD2* allele (NOD2-WT), was lost in patients homozygous for the *NOD2 3020insC* mutation. Medium-stimulated cells resulted in cytokine concentrations below detection limit. Data are presented as mean ± SD (*p < 0.05).

In order to study the cross talk between TLR2 and NOD2 in the recognition of *M. tuberculosis,* we investigated whether mycobacterial TLR2 and NOD2 ligands synergize for the induction of cytokine release. Indeed, the NOD2 ligand MDP, the minimal component of peptidoglycan responsible for NOD2 activation, had strong synergistic effect on TNF production induced by the 19-kDa lipoprotein of *M. tuberculosis,* a specific mycobacterial TLR2 ligand (Figure 6A). Similar synergistic effects between the 19-kDa lipoprotein and MDP were observed when IL-6 (4.5-fold synergism), and IL-1β (7.2-fold synergism) were measured. This synergism was lost in individuals homozygous for the NOD2 *3020insC* mutation (Figure 6B) or macrophages harvested from TLR2^−/−^ mice (5.7-fold synergism between lipoprotein 19-kDa and FK-156 in control macrophages, and 1.9-fold synergism in TLR2^−/−^ cells). No such synergy was observed between MDP and mannosyl-caped lipoarabinomannan, a component of *M. tuberculosis* that does not interact with TLR2 (unpublished data).

## Discussion

In the present study, we investigated the role of TLRs and NODs, the two most important classes of PRRs in the recognition by macrophages of *M. tuberculosis*. Although we confirmed the role of TLR2 and TLR4 for in mycobacterial recognition, strong residual activity was detectable in cells lacking TLR2, which suggests the existence of TLR-independent recognition mechanisms; this idea is supported by a study demonstrating MyD88-independent pathways of macrophage stimulation by *M. tuberculosis* [22]. Using cell lines transfected with *NOD1* and *NOD2*, as well as primary MNCs defective in these receptors, we demonstrated that, in addition to TLRs, NOD2 represents a nonredundant recognition system of *M. tuberculosis.* We also demonstrated that mycobacterial TLR2 and NOD2 ligands synergize for the production of proinflammatory cytokines, and that this synergism is lost in cells lacking either of these receptors.

Several studies have demonstrated the role of TLR2 and TLR4 in the recognition of *M. tuberculosis*. The 19-kDa lipoprotein [4], lipomannan [5], and phosphatidyl-myo-inositol mannoside [6], all components of mycobacteria, have been identified as being responsible for TLR2-dependent activation, whereas heat-labile components associated with the cell wall were found to signal via TLR4 [3]. A role for TLRs in antimycobacterial defense was also suggested by the enhanced susceptibility to *M. tuberculosis* infection in mice deficient for MyD88, an adapter molecule shared by almost all TLR family members [15]. Similarly, TLR2^−/−^ mice had a decreased clearance of the bacteria and developed chronic pneumonia when infected with low doses of microorganisms [9,13,14], whereas TLR4^−/−^ mice showed variable responses to challenge with *M. tuberculosis* [9,10].

Our data confirm the important role played by TLRs, and especially TLR2, in the recognition of *M. tuberculosis,* but at the same time demonstrate strong TLR-independent induction of cytokines by this microorganism. The contribution of TLR4 was found to be less crucial in our study, and could be observed only when cells were stimulated with intact microorganisms.

Because *M. tuberculosis* is an intracellular pathogen, we hypothesized that intracellular recognition systems could contribute to the sensing of mycobacteria and stimulation of innate immunity. To test this hypothesis, we studied the effect of blocking the internalization of *M. tuberculosis* with cytochalasin B. Blocking internalization of *M. tuberculosis* partially inhibited *M. tuberculosis-*induced cytokine release in MNCs, whereas cytochalasin B potentiated the cytokine induction by zymosan, likely due to prolonged stimulation of receptors at the cell surface by zymosan. The differential effects of cytochalasin B on *M. tuberculosis-* or zymosan-induced cytokine secretion demonstrate that, in addition to the interaction with cell-membrane bound TLRs, *M. tuberculosis* is recognized by and induces cytokine production through intracellular receptors.

NOD2 and NOD1 are members of the expanding CATERPILLER family of proteins, which share an LRR domain similar to that found in TLRs [23]. NOD2 has been linked genetically to increased risk for Crohn's disease [16,17], and is a sensor of bacterial peptidoglycans [18]. When HEKs transfected with either *NOD1* or *NOD2* were stimulated with *M. tuberculosis* cell wall preparations, both of them—but most markedly those transfected with *NOD2*—showed a dose-dependent response. These data are consistent with the conclusion that NOD2 is a general sensor of bacteria through the detection of MDP, a peptidoglycan substructure present in bacterial cell walls [18]. However, the fact that NOD1 was found to be a poor sensor of *M. tuberculosis* not only in *NOD1*-transfected HEK cells, but also through the lack of a defective response in NOD1^−/−^ macrophages, is somewhat puzzling. Indeed, NOD1 detects diaminopimelic acid (DAP)-type peptidoglycans, and earlier reports had suggested that *M. tuberculosis* peptidoglycan is of this category. Further investigation is therefore required to discover why NOD1 detects *M. tuberculosis* poorly.

To test whether the data in transfected cell lines could be reproduced in primary cells, we stimulated MNCs isolated from Crohn's disease patients homozygous for the *3020insC* null-mutant allele with *M. tuberculosis*. This mutation leads to the deletion of the last 32 amino acids of the LRR region responsible for the detection of peptidoglycan, and we have recently shown that cells isolated from these patients are completely unable to recognize MDP or gram-positive peptidoglycan [21]. In line with the hypothesis that NOD2 is involved in the recognition of mycobacteria, both peritoneal macrophages from NOD2-deficient mice and MNCs isolated from patients homozygous for the *3020insC* mutation of *NOD2* were found to synthesize significantly less cytokines after stimulation with *M. tuberculosis*. Interestingly, the very strong defect in the response to *M. tuberculosis* of the cells of patients with the *NOD2 3020insC* mutation suggests that NOD1 is not able to compensate for the defective NOD2 recognition. This is in line with the weak stimulation of *NOD1*-transfected HEKs by *M. tuberculosis* and our recent finding that NOD2 is needed for normal signaling by NOD1 ligands such as Mur-Tri-DAP [24]. The reverse is not true in the case of NOD1: Macrophages harvested from NOD1^−/−^ mice responded normally to *M. tuberculosis,* demonstrating that the absence of NOD1 can be compensated by other recognition systems, most likely NOD2.

The finding of strongly reduced cytokine production after stimulation with *M. tuberculosis* in cells of patients with a defective NOD2 and of NOD2 knock-out mice demonstrates that NOD2 is a key sensor of *M. tuberculosis* in mammalian cells. Interestingly, inhibition of both NOD2 and TLR2 systems blocked stimulation of cytokines by *M. tuberculosis* by substantially more than 50%. This suggests that the signaling pathways induced by these receptors interact and potentiate each other, and, conversely, that defects in one pathway lead to a loss of synergy. We have recently shown that NOD2 signals strongly synergize with specific TLR pathways such as TLR2, TLR4, and TLR3 [25]. We therefore investigated whether NOD2 activation leads to similar synergistic cytokine stimulation by TLR2 ligands specifically derived from *M. tuberculosis*. Indeed, MDP had strong synergistic effect on the cytokine production induced by the 19-kDa lipoprotein of *M. tuberculosis,* and this synergy was lost both in individuals homozygous for the *NOD2 3020insC* mutation and in macrophages harvested from TLR2^−/−^ mice.

An issue yet to be resolved is represented by the mechanism through which mycobacterial peptidoglycans come in contact with NOD2. NOD2 is an intracytoplasmic molecule, while *M. tuberculosis* remains located mainly in phagosomes. Although shedding of cell wall components from the microorganism is likely responsible for the release of peptidoglycans that are ultimately recognized by NOD2, the precise mechanism through which peptidoglycans translocate from the phagosome into the cytoplasm remains to be identified.

The data presented in this study demonstrate that NOD2 and TLRs are two nonredundant recognition mechanisms of *M. tuberculosis*. Both are essential for effective activation of the proinflammatory cytokine production by *M. tuberculosis,* and they strengthen each other's activity through synergistic effects. This demonstrates that host cells sense the presence of *M. tuberculosis* using multiple recognition systems in which different classes of receptors, in this case NOD2 and TLRs, interact with each other.

The involvement of NOD2 in the recognition of *M. tuberculosis* has several implications. First, it is possible that NOD2 is involved in recognition of other gram-positive bacteria with cell walls rich in peptidoglycans. The recent report of NOD2 serving as a receptor for *Streptococcus pneumoniae* supports this idea [26]. Second, our data suggest that NOD2 is involved in the recognition of other *Mycobacteria* species. From this point of view, *M. paratuberculosis* is of particular interest, due to its possible involvement in the pathogenesis of Crohn's disease [27]. A defective host defense against *M. paratuberculosis* in individuals bearing loss-of-function *NOD2* mutations may be responsible for the invasion of the intestine and may promote chronic inflammation ultimately leading to Crohn's disease. This hypothesis is supported by the recently described presence of *M. paratuberculosis* in the circulation of patients with Crohn's disease [28]. Regarding the role of NOD2 in tuberculosis, one must, however, acknowledge that the present study provides information only on the in vitro recognition of *M. tuberculosis,* and studies investigating the role of NOD2 in infection models are warranted.

In conclusion, NOD2 and TLRs appear to serve as independent, nonredundant PRRs of *M. tuberculosis.* The intracellular pathways induced by NOD2 and TLR2 during recognition of mycobacterial components synergize, and the stimulation of cytokine production by *M. tuberculosis* is greatly impaired in individuals with *NOD2* mutations.

## Materials and Methods

### Reagents and microorganisms.

Synthetic Pam3Cys and 19-kDa lipoprotein were purchased from EMC Microcollections (Tübingen, Germany). MDP and LPS (*E. coli* serotype 055:B5) were purchased from Calbiochem (San Diego, California, United States) and Sigma (St. Louis, Missouri, United States), respectively. The synthetic MDP was tested for contamination with lipoproteins or LPS in mice deficient in TLR2 or TLR4, respectively. No defect in cytokine production was apparent in these mice after stimulation with MDP, demonstrating the absence of contamination.

Cultures of *M. tuberculosis* H37Rv were grown to mid-log phase in Middlebrook 7H9 liquid medium supplemented with oleic acid/albumin/dextrose/catalase (Difco, Becton-Dickinson, Palo Alto, California, United States), washed three times in sterile saline, and resuspended in RPMI 1640 medium at the various concentrations. Separate culture suspensions were sonicated for 10 min on ice, in order to obtain cell lysates.

### Genotyping of *NOD2* variants.

Blood was collected from 74 patients with Crohn's disease and ten healthy volunteers. PCR amplification of *NOD2* gene fragments containing the polymorphic site *3020insC* was performed in 50-μl reaction volumes containing 100–200 ng of genomic DNA as previously described [21]. The *3020insC* polymorphism was analyzed by Genescan analysis on an ABI-Prism 3100 Genetic Analyzer according to the protocol of the manufacturer (Applied Biosystems, Nieuwerkerk a/d IJssel, The Netherlands).

Four patients with Crohn's disease were found to be homozygous for the *3020insC* mutation, and they were further investigated in the cytokine studies. As control groups, five patients with Crohn's disease who were heterozygous for the *3020insC NOD2* mutation, five patients with Crohn's disease bearing the wild-type allele, and five healthy volunteers homozygous for the wild-type *NOD2* allele were included. None of the patients with Crohn's disease used immunosuppressive medication at the time of the study.

### Isolation of MNCs and stimulation of cytokine production.

After informed consent, venous blood was drawn from the cubital vein of patients and healthy volunteers into three 10-ml EDTA tubes (Monoject, s-Hertogenbosch, The Netherlands). Isolation of MNCs was performed as described elsewhere [29], with minor modifications. The MNC fraction was obtained by density centrifugation of blood diluted 1:1 in pyrogen-free saline over Ficoll-Paque (Pharmacia Biotech AB, Uppsala, Sweden). Cells were washed twice in saline and suspended in culture medium (RPMI 1640 DM) supplemented with 10 μg/ml gentamicin, 10 mM L-glutamine, and 10 mM pyruvate. The cells were counted in a Coulter counter (Coulter Electronics, Mijdrecht, The Netherlands), and the number was adjusted to 5 × 10^6^ cells/ml.

MNCs (5 × 10^5^) were added in a 100-μl volume to round-bottom 96-well plates (Greiner, Alphen a/d Rijn, The Netherlands) and incubated with either 100 μl of culture medium (negative control), or the various stimuli: *M. tuberculosis* bacteria (1 × 10^5^ to 1 × 10^7^ microorganisms/ml), *M. tuberculosis* sonicate (10 μg/ml), MDP (1 μg/ml), LPS (10 ng/ml), 19-kDa lipoprotein (concentrations as described below), or combinations of MDP and 19-kDa lipoprotein. The influence of internalization of *M. tuberculosis* on cytokine production was investigated by adding 20 μg/ml cytochalasin B during stimulation with the microorganism. Positive control stimulation for the effects of cytochalasin B was provided by stimulation of cells with zymosan (1 μg/ml; Sigma). All stimuli were checked for the contamination with LPS in the Limulus amoebocyte lysate assay and found to be negative. Cytochalasin B did not influence cell viability (unpublished data). To evaluate the role of TLR4 in the induction of cytokines, cells were preincubated with 10 μg/ml of a blocking monoclonal anti-TLR4 antibody (eBioscience, San Diego, California, United States).

### Cytokine production by murine peritoneal macrophages.

Resident peritoneal macrophages from either ScCr (TLR4-defective) or C57Bl/10J (TLR4 control) mice, TLR2^−/−^ or control TLR2^+/+^ mice (kindly provided by S. Akira, Osaka, Japan) [30], NOD1^−/−^ and NOD1^+/+^ littermates (kindly provided by E. Abraham, Denver, Colorado, United States), or NOD2^+/+^ and NOD2^−/−^ (from M. Giovannini, CEPH, Paris), backcrossed to the seventh generation into the C57BL6/J background, were harvested by injection of 4 ml of sterile PBS containing 0.38% sodium citrate [31]. After centrifugation and washing, the cells were resuspended in RPMI 1640 containing 1 mM pyruvate, 2 mM L-glutamine, 100 μg/ml gentamicin, and 2% fresh mouse plasma. Cells were cultured in 96-well microtiter plates (Greiner) at 1 × 10^5^ cells/well, in a volume of 100 μl. The cells were stimulated with purified LPS (1 μg/ml), Pam3Cys (1 μg/ml), FK-156 ligand of murine NOD1 (1 μg/ml) as a control stimulation, or a sonicate of *M. tuberculosis* (1 μg/ml). After 24 h incubation at 37 °C, the supernatants were collected and stored at −70 °C until cytokine assays were performed.

### Cytokine measurements.

Human and murine TNFα concentrations were determined by specific RIAs as described [32,33]. IL-10 and IL-6 were measured by a commercial ELISA kits (Pelikine Compact, CLB, Amsterdam, The Netherlands), according to the instructions of the manufacturer.

### Signaling through human TLR2 and TLR4 in transfected cell lines.

CHOs stably transfected with human CD14 (3E10-CD14), a combination of CD14 and TLR2 (3E10-TLR2), or TLR4 (3E10-TLR4), were a kind gift from R. Ingalls [34]. All cell lines express inducible membrane CD25 under control of a region from the human E-selectin (ELAM-1) promoter containing NF-κB binding sites. Cells were maintained at 37 °C and 5% CO_2_ in HAM's F12 medium (Gibco, Invitrogen, Breda, The Netherlands) supplemented with 10% FCS, 0.01% L-glutamine, 50 μg/ml gentamicin, and either 400 U/ml hygromycin and 0.5 mg/ml of G418 (for 3E10-TLR2) or 0.05 mg/ml of puromycin (for 3E10-TLR4) as additional selection antibiotics. TLR2 and TLR4 expression was confirmed by flow cytometry (Coulter Epics XL-MCL, Beckman Coulter, Mijdrecht, the Netherlands) using PE-labeled anti-TLR2 (clone TL2.1) or anti-TLR4 (clone HTA125) (Immunosource, Halle-Zoersel, Belgium).

For stimulation experiments, 500 μl of cells in culture medium at a density of 1 × 10^5^/ml were plated in 24-well culture plates. After an overnight incubation, cells were incubated with control medium, *M. tuberculosis* sonicate (10 μg/ml), Pam3Cys (10 μg/ml), or LPS (1 μg/ml) for 20 h, and thereafter cells were harvested using trypsin/EDTA (Cambrex, East Rutherford, New York, United States) and prepared for flow cytometry (Coulter FACS-scan). CD25 expression of the CHOs was measured using FITC-labeled anti-CD25 (DAKO, Glostrup, Denmark), and expressed as folds-over-mean increase.

### Stimulation of NOD-transfected cell lines and NF-κB translocation.

Studies examining the activation of NF-κB by *M. tuberculosis* in cells overexpressing NOD1 or NOD2 were carried out as previously described [19]. Briefly, 1 × 10^6^/ml HEK293T cells were transfected overnight with 1 ng of either NOD1 or NOD2 plus 75 ng luciferase reporter plasmid. At the same time, heat-killed whole *M. tuberculosis* preparations (ratio of microorganisms and effector cells 1:10, 1:1, and 10:1) were added to cell culture medium, and the NF-κB–dependent luciferase activation was then measured following 24 h of incubation. NF-κB–dependent luciferase assays were performed in duplicate, and data represent three independent experiments [19].

### Statistical analysis.

The human experiments were performed in triplicate with blood obtained from patients and volunteers. The mouse experiments were performed twice in 10 mice per group, and the data are presented as cumulative results of all experiments performed. The differences between groups were analyzed by unpaired Student t-test, and where appropriate by paired t-test. The level of significance between groups was set at *p* < 0.05. The data are given as means ± standard deviation (SD).

## Abbreviations

CHO: Chinese hamster ovary fibroblast
HEK293T: human embryonic kidney 293T cell
LPS: lipopolysaccharide
LRR: leucine-rich repeat
MDP: muramyl dipeptide
MNC: mononuclear cell
NOD: nucleotide-binding oligomerization domain
PRR: pattern recognition receptor
SD: standard deviation
TLR: Toll-like receptor
TNF: tumor necrosis factor

## References

[ppat-0010034-b001] Frieden TR, Sterling TR, Munsiff SS, Watt CJ, Dye C (2003). Tuberculosis. Lancet.

[ppat-0010034-b002] Van Crevel R, Ottenhoff TH, van der Meer JW (2002). Innate immunity to *Mycobacterium tuberculosis*. Clin Microbiol Rev.

[ppat-0010034-b003] Means TK, Wang S, Lien E, Yoshimura A, Golenbock DT (1999). Human Toll-like receptors mediate cellular activation by *Mycobacterium tuberculosis*. J Immunol.

[ppat-0010034-b004] Aliprantis AO, Yang R-B, Mark MR, Sugget S, Devaux B (1999). Cell activation and apoptosis by bacterial lipoproteins through Toll-like receptor-2. Science.

[ppat-0010034-b005] Vignal C, Guerardel Y, Kremer L, Masson M, Legrand D (2003). Lipomannans, but not lipoarabinomannans, purified from *Mycobacterium chelonae* and *Mycobacterium kansasii* induce TNF-alpha and IL-8 secretion by a CD14-Toll-like receptor-2 -dependent mechanism. J Immunol.

[ppat-0010034-b006] Gilleron M, Quesniaux VF, Puzo G (2003). Acylation state of the phosphatidylinositol hexamannosides from *Mycobacterium bovis* bacillus Calmette Guérin and *Mycobacterium tuberculosis* H37Rv and its implication in Toll-like receptor response. J Biol Chem.

[ppat-0010034-b007] Nigou J, Zelle-Rieser C, Gilleron M, Thurnher M, Puzo G (2001). Mannosylated lipoarabinomannans inhibit IL-12 production by human dendritic cells: Evidence for a negative signal delivered through the mannose receptor. J Immunol.

[ppat-0010034-b008] Geijtenbeek TB, Van Vliet SJ, Koppel EA, Sanchez-Hernandez M, Vanderbroucke-Grauls CM (2003). Mycobacteria target DC-SIGN to suppress dendritic cell function. J Exp Med.

[ppat-0010034-b009] Reiling N, Holscher C, Fehrenbach A, Svenja K, Kirschning CJ (2002). Toll-like receptor (TLR)2- and TLR4-mediated pathogen recognition in resistance to airborne infection with *Mycobacterium tuberculosis*. J Immunol.

[ppat-0010034-b010] Kamath AB, Alt J, Debbabi H, Behar SM (2003). Toll-like receptor-4 defective C3H/HeJ mice are not more susceptible than other C3H substrains to infection with *Mycobacterium tuberculosis*. Infect Immun.

[ppat-0010034-b011] Abel B, Thieblemont N, Quesniaux VF, Brown N, Mpagi J (2002). Toll-like receptor 4 expression is required to control chronic *Mycobacterium tuberculosis* infection in mice. J Immunol.

[ppat-0010034-b012] Branger J, Leemans JC, Florquin S, Weijer S, Speelman P (2004). Toll-like receptor 4 plays a protective role in pulmonary tuberculosis in mice. Int Immunol.

[ppat-0010034-b013] Drennan MB, Nicolle D, Quesniaux VF, Jacobs M, Allie N (2004). Toll-like receptor 2 deficient mice succumb to *Mycobacterium tuberculosis* infection. Am J Pathol.

[ppat-0010034-b014] Sugawara I, Yamada H, Li C, Mizuno S, Takeuchi O (2003). Mycobacterial infection in TLR2 and TLR6 knockout mice. Microbiol Immunol.

[ppat-0010034-b015] Fremond C, Yeremeev V, Nicolle DM, Jacobs M, Quesniaux VF (2004). Fatal *Mycobacterium tuberculosis* infection despite adaptive immune response in the absence of MyD88. J Clin Invest.

[ppat-0010034-b016] Hugot J-P, Chamaillard M, Zouali H, Lesage S, Cezard J-P (2001). Association of NOD2 leucine-rich repeat variants with susceptibility to Crohn's disease. Nature.

[ppat-0010034-b017] Ogura Y, Bonen DK, Inohara N, Nicolae L, Chen FF (2001). A frameshift mutation in NOD2 associated with susceptibility to Crohn's disease. Nature.

[ppat-0010034-b018] Girardin SE, Boneca IG, Viala J, Chamaillard M, Labigne A (2003). Nod2 is a general sensor of peptidoglycan through muramyl dipeptide (MDP) detection. J Biol Chem.

[ppat-0010034-b019] Girardin SE, Boneca IG, Carneiro LAM, Antignac A, Jehanno M (2003). Nod1 detects a unique muropeptide from gram-negative bacterial peptidoglycan. Science.

[ppat-0010034-b020] Quesniaux VF, Fremond C, Jacobs M, Parida S, Nicolle D (2004). Toll-like receptor pathways in the immune responses to mycobacteria. Microbes Infect.

[ppat-0010034-b021] Netea MG, Kullberg BJ, de Jong D, Francke B, Sprong T (2004). NOD2 mediates induction of the antiinflammatory signals induced by TLR2-ligands: Implications for Crohn's disease. Eur J Immunol.

[ppat-0010034-b022] Shi S, Nathan C, Schnappinger D, Drenkow J, Fuortes M (2003). MyD88 primes macrophages for full-scale activation by interferon-gamma yet mediates few responses to *Mycobacterium tuberculosis*. J Exp Med.

[ppat-0010034-b023] Ting JP-Y, Davis BK (2005). CATERPILLER: A novel gene family important in immunity, cell death, and disease. Annu Rev Immunol.

[ppat-0010034-b024] Netea MG, Ferwerda G, De Jong DJ, Werts C, Boneca IG (2005). The frameshift mutation in Nod2 results in unresponsiveness not only to Nod2-, but also Nod1-activating peptidoglycan agonists. J Biol Chem.

[ppat-0010034-b025] Netea MG, Ferwerda G, De Jong DJ, Jansen T, Jacobs L (2005). NOD2 modulates specific Toll-like receptor pathways for the induction of cytokine release. J Immunol.

[ppat-0010034-b026] Opitz B, Puschel A, Schmeck B, Hocke AC, Rosseau S (2004). Nucleotide-binding oligomerization domain proteins are innate immune receptors for internalized *Streptococcus pneumoniae*. J Biol Chem.

[ppat-0010034-b027] Greenstein RJ (2003). Is Crohn's disease caused by a mycobacterium? Comparisons with leprosy, tuberculosis, and Johne's disease. Lancet Infect Dis.

[ppat-0010034-b028] Naser SA, Ghobrial G, Romero C, Valentine JF (2004). Culture of *Mycobacterium avium* subspecies *paratuberculosis* from the blood of patients with Crohn's disease. Lancet.

[ppat-0010034-b029] Endres S, Ghorbani R, Lonnemann G, Van der Meer JWM, Dinarello CA (1988). Measurement of immunoreactive interleukin-1 beta from human mononuclear cells: Optimization of recovery, intrasubject consistency, and comparison with interleukin-1 alpha and tumor necrosis factor. Clin Immunol Immunopathol.

[ppat-0010034-b030] Takeuchi O, Hoshino K, Kawai T, Sanjo H, Takada H (1999). Differential roles of TLR2 and TLR4 in recognition of gram-negative and gram-positive bacterial cell wall components. Immunity.

[ppat-0010034-b031] Kullberg BJ, Van't Wout JW, Hoogstraten C, Van Furth R (1993). Recombinant interferon-γ enhances resistance to acute disseminated *Candida albicans* infection in mice. J Infect Dis.

[ppat-0010034-b032] Drenth JPH, Van Uum SHM, Van Deuren M, Pesman GJ, Van der Ven-Jongekrijg J (1995). Endurance run increases circulating IL-6 and IL-1ra but downregulates ex vivo TNF-α and IL-1β production. J Appl Physiol.

[ppat-0010034-b033] Netea MG, Demacker PNM, Kullberg BJ, Boerman OC, Verschueren I (1996). Low-density-lipoprotein receptor deficient mice are protected against lethal endotoxinemia and severe gram-negative infections. J Clin Invest.

[ppat-0010034-b034] Lien E, Means TK, Heine H, Yoshimura A, Kusumoto S (2000). Toll-like receptor 4 imparts ligand-specific recognition of bacterial lipopolysaccharide. J Clin Invest.

